# Supplementary figures and images for: Simulating Metabolic Flexibility in Low Energy Expenditure Conditions Using Genome-Scale Metabolic Models
Source: Metabolites. 2021 Oct 12;11(10):695. doi: 10.3390/metabo11100695 (PMC8537358; doi:10.3390/metabo11100695)

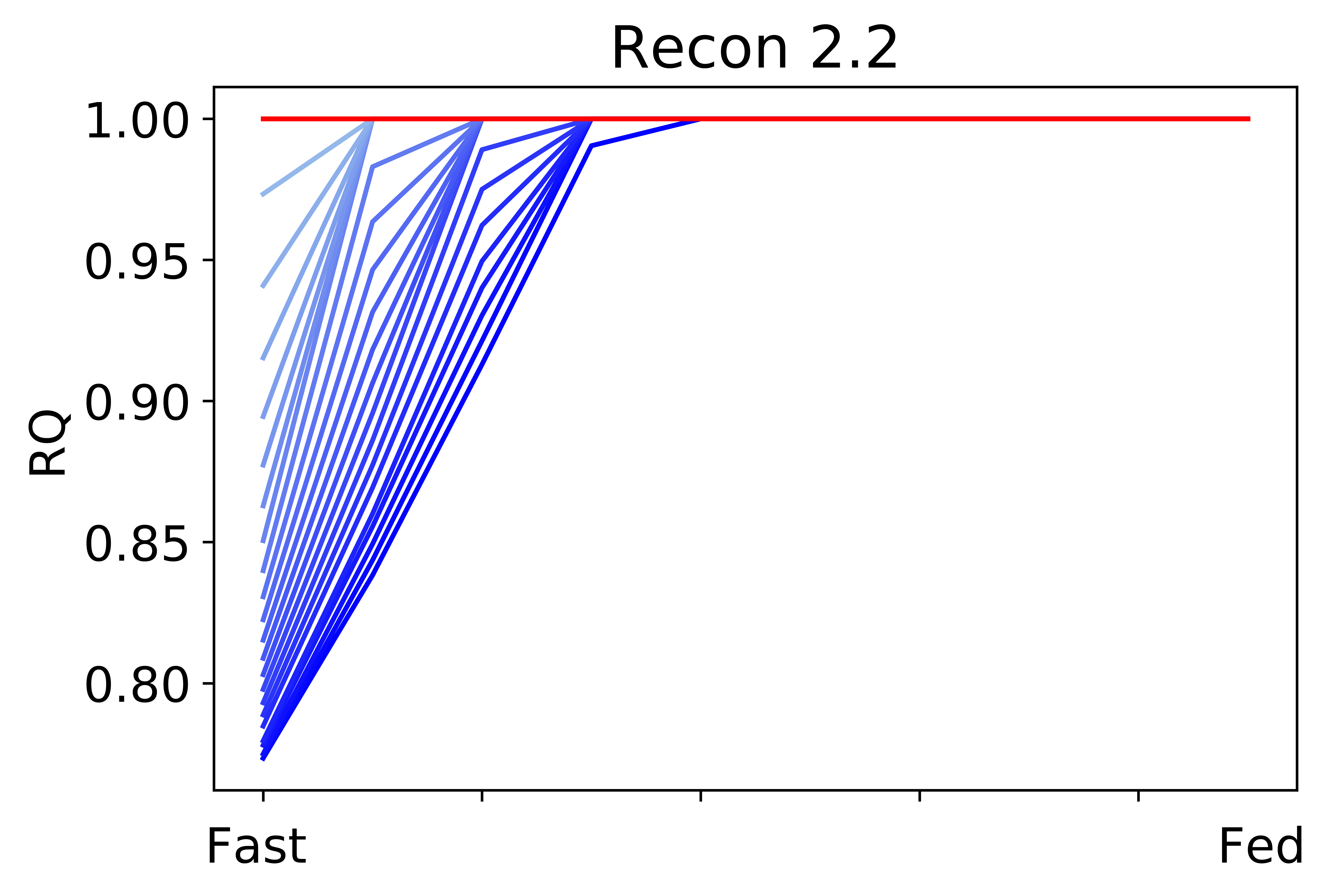

Supplement: Supplementary file 1 [file metabolites-11-00695-s001.zip › supplementary figures/figureS2A.png]

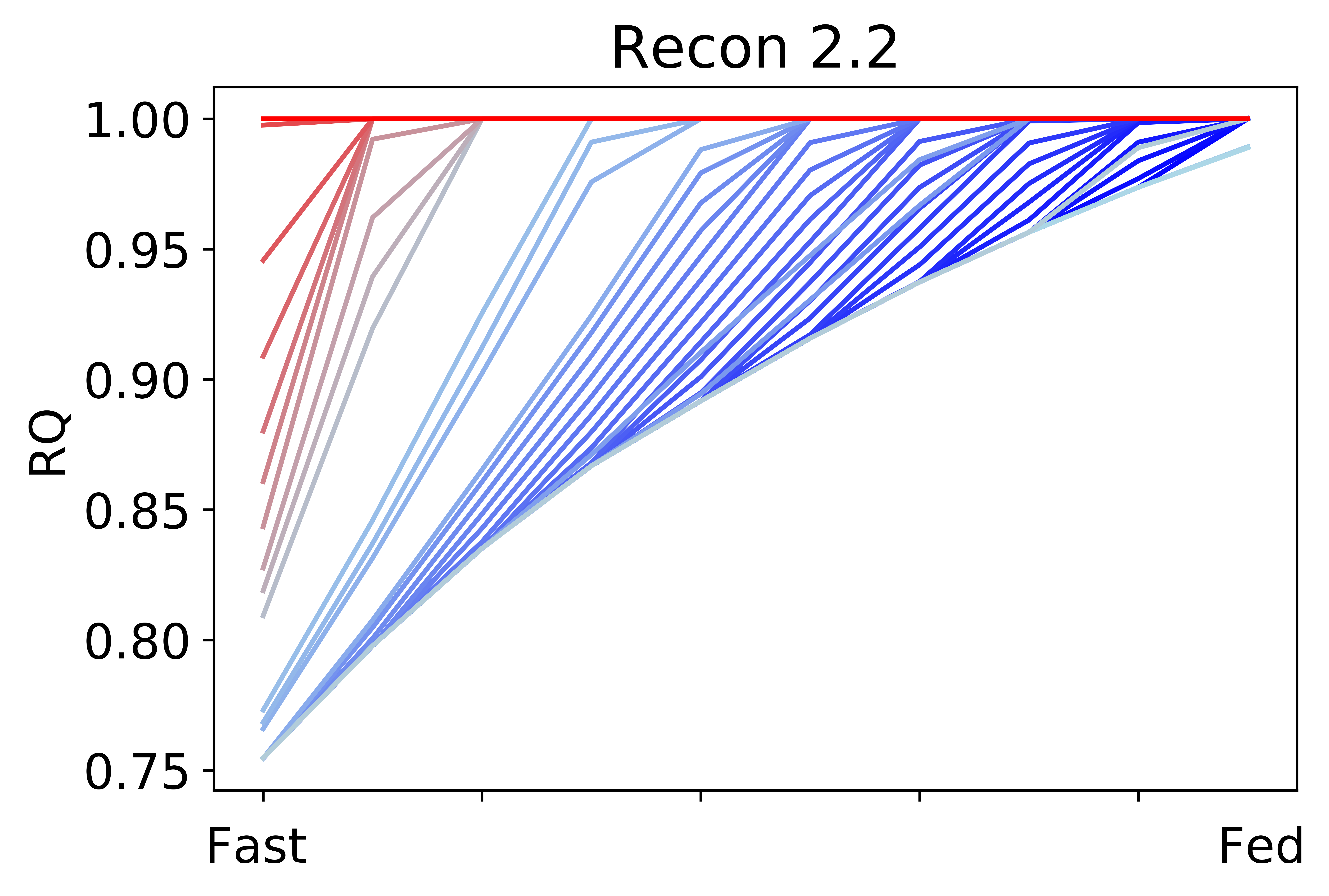

Supplement: Supplementary file 1 [file metabolites-11-00695-s001.zip › supplementary figures/figure3A.png]

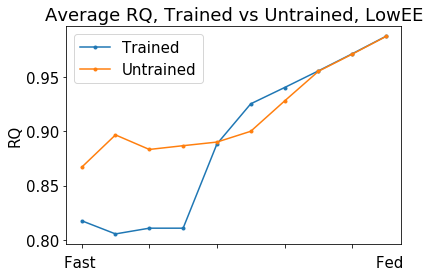

Supplement: Supplementary file 1 [file metabolites-11-00695-s001.zip › supplementary figures/figure4B.png]

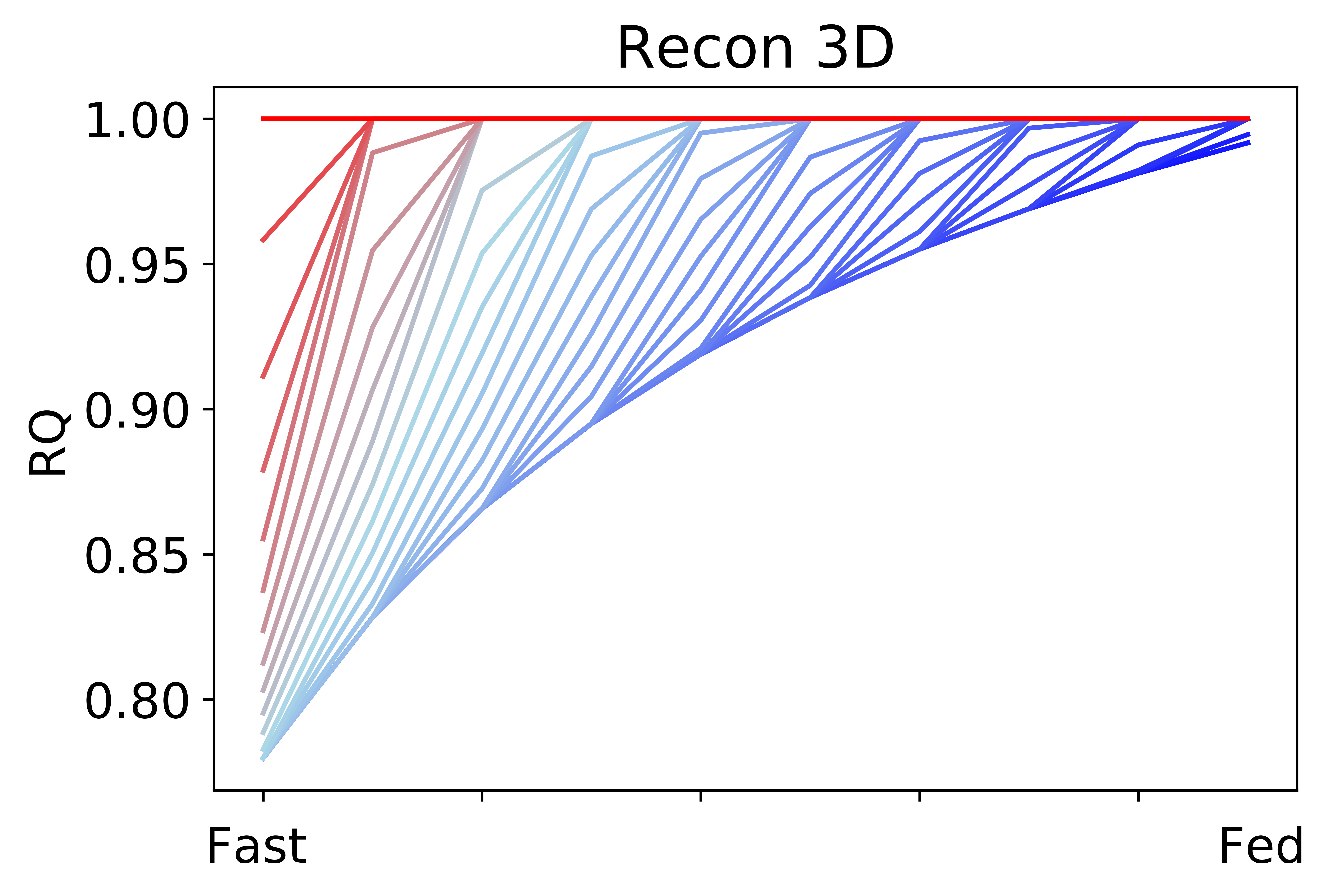

Supplement: Supplementary file 1 [file metabolites-11-00695-s001.zip › supplementary figures/figure3B.png]

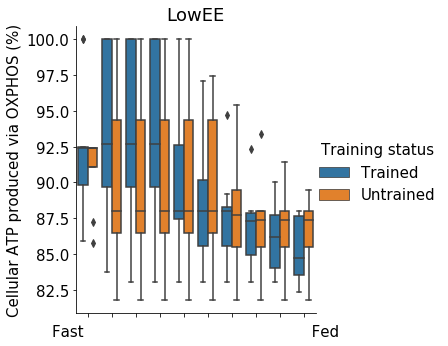

Supplement: Supplementary file 1 [file metabolites-11-00695-s001.zip › supplementary figures/figure5B.png]

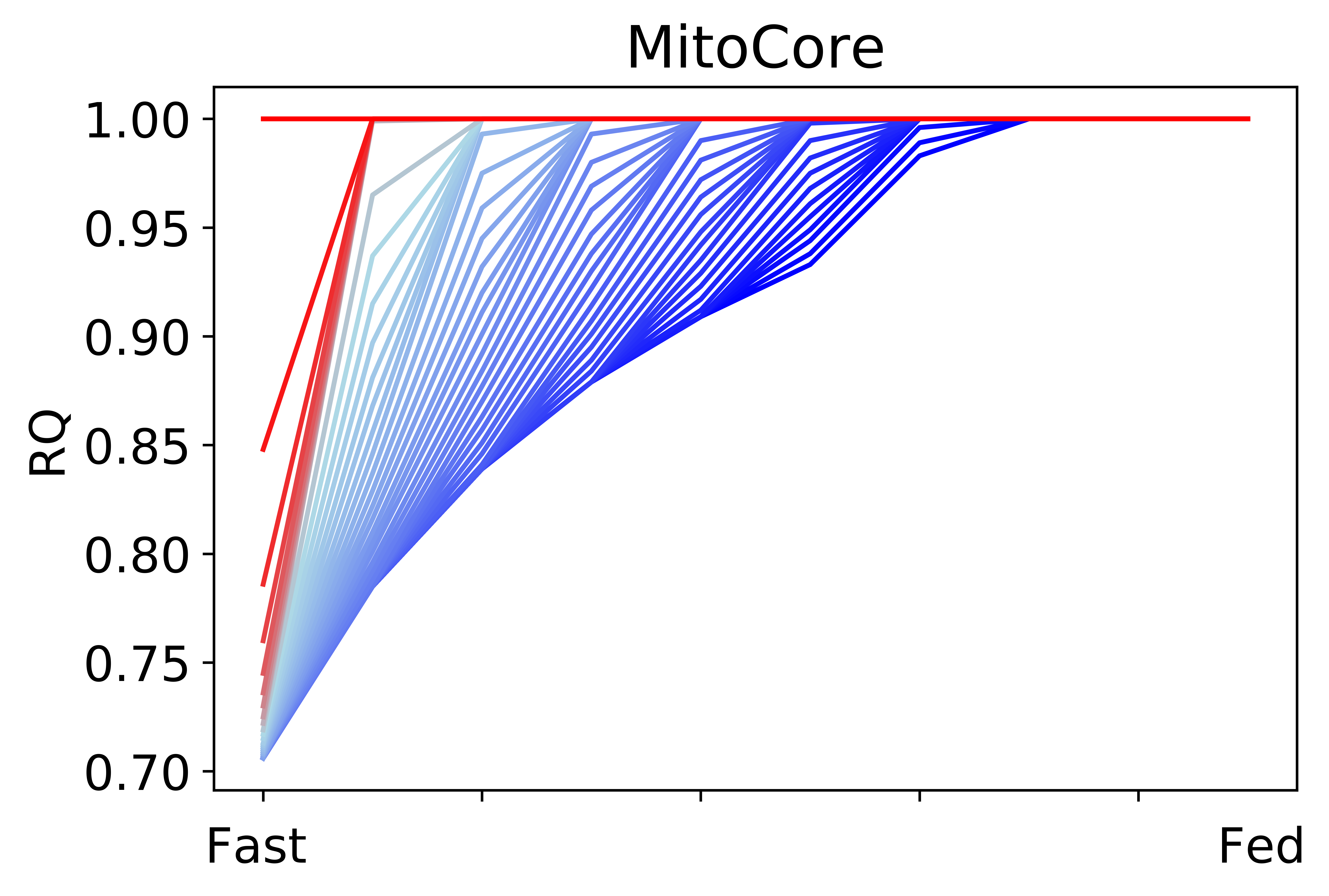

Supplement: Supplementary file 1 [file metabolites-11-00695-s001.zip › supplementary figures/figure3C.png]

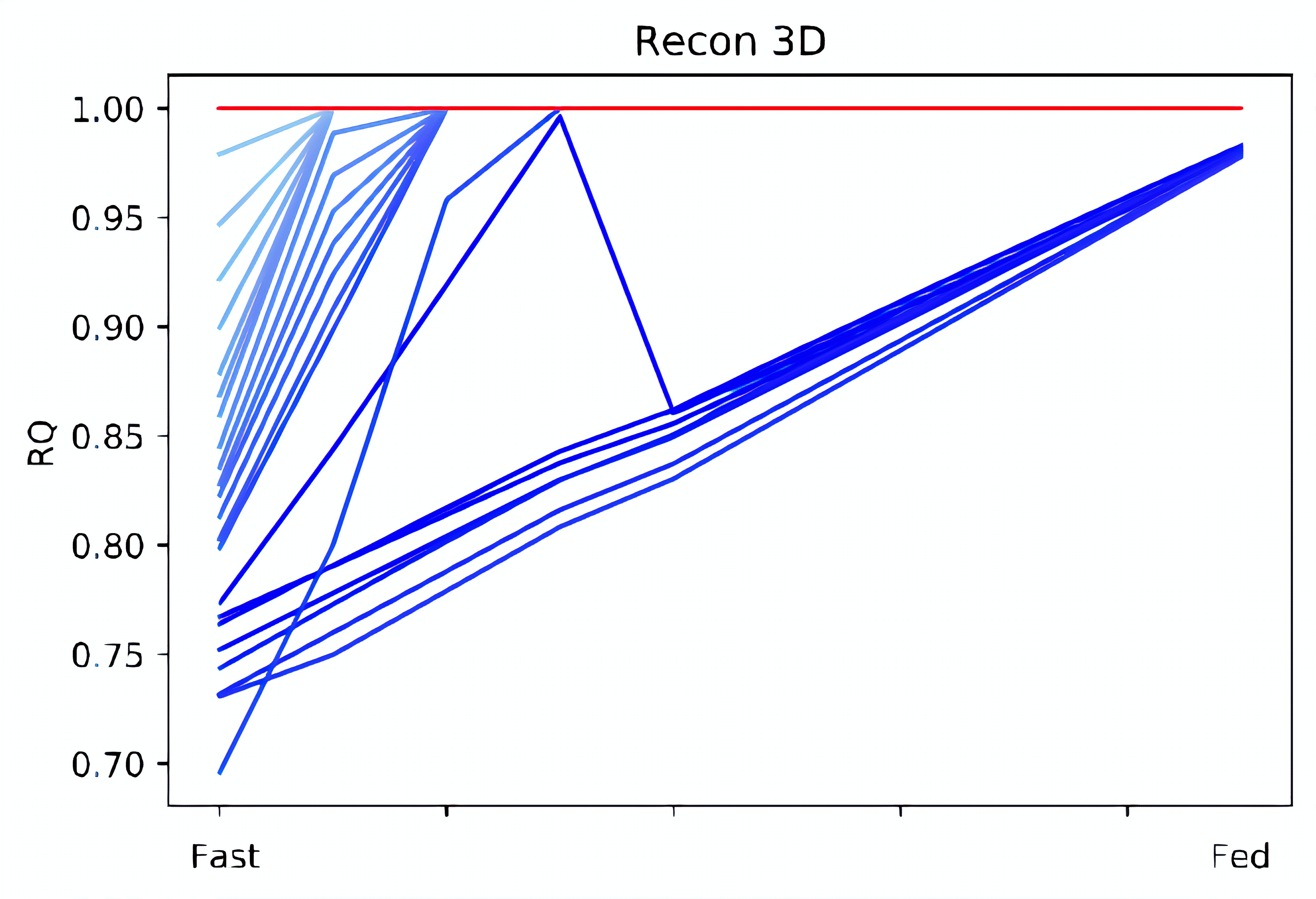

Supplement: Supplementary file 1 [file metabolites-11-00695-s001.zip › supplementary figures/figureS2B.png]

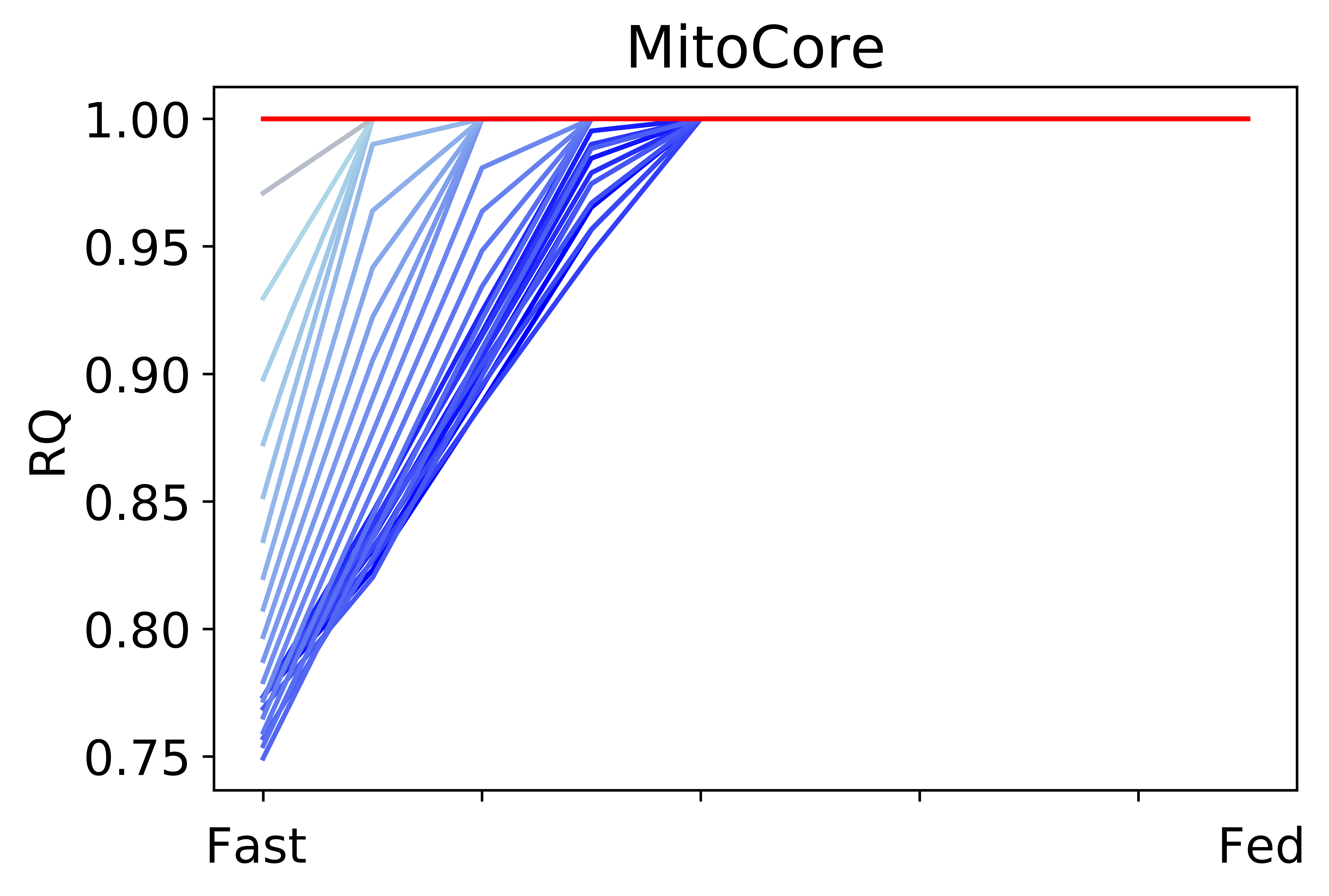

Supplement: Supplementary file 1 [file metabolites-11-00695-s001.zip › supplementary figures/figureS2C.png]

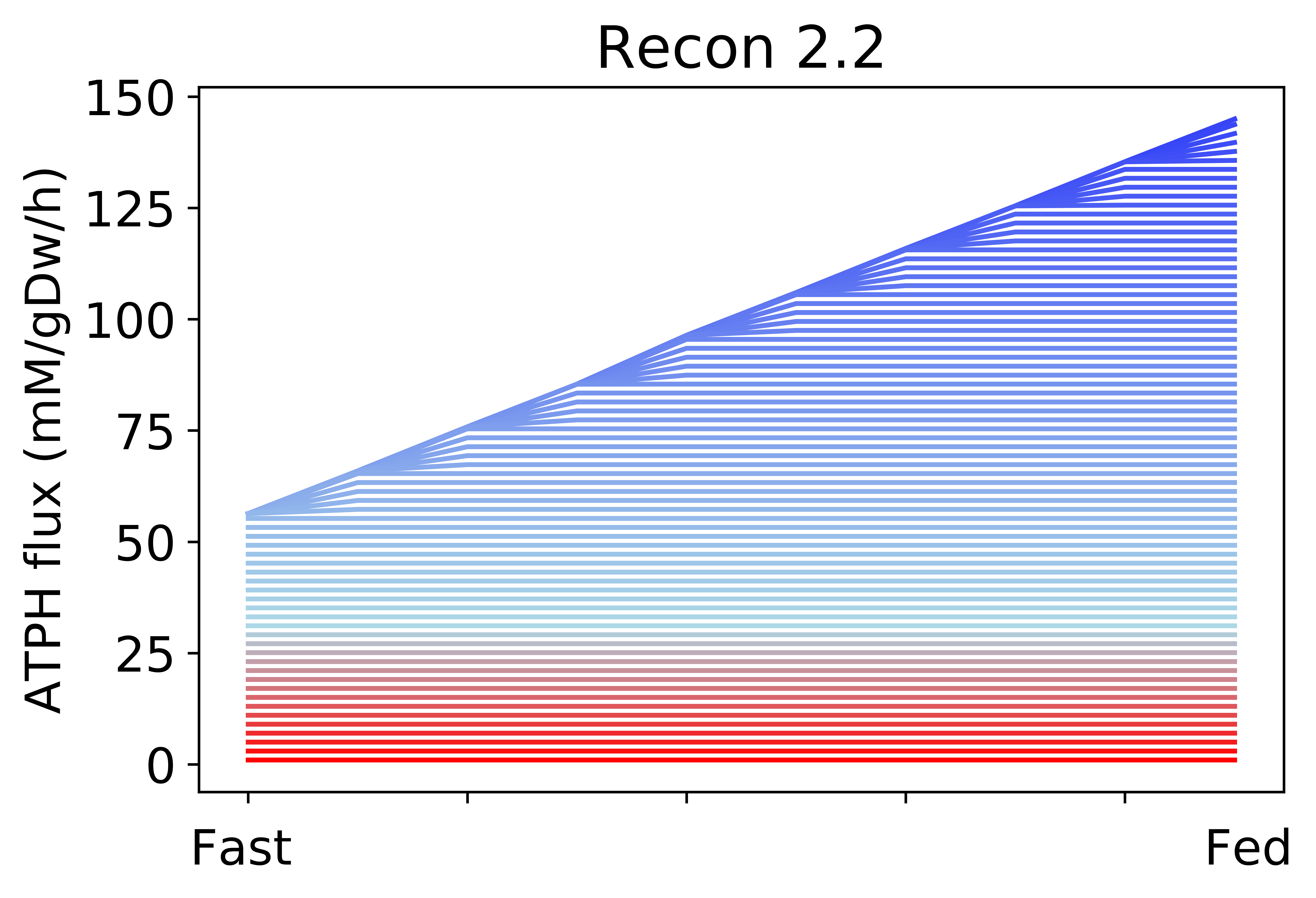

Supplement: Supplementary file 1 [file metabolites-11-00695-s001.zip › supplementary figures/figureS3A.png]

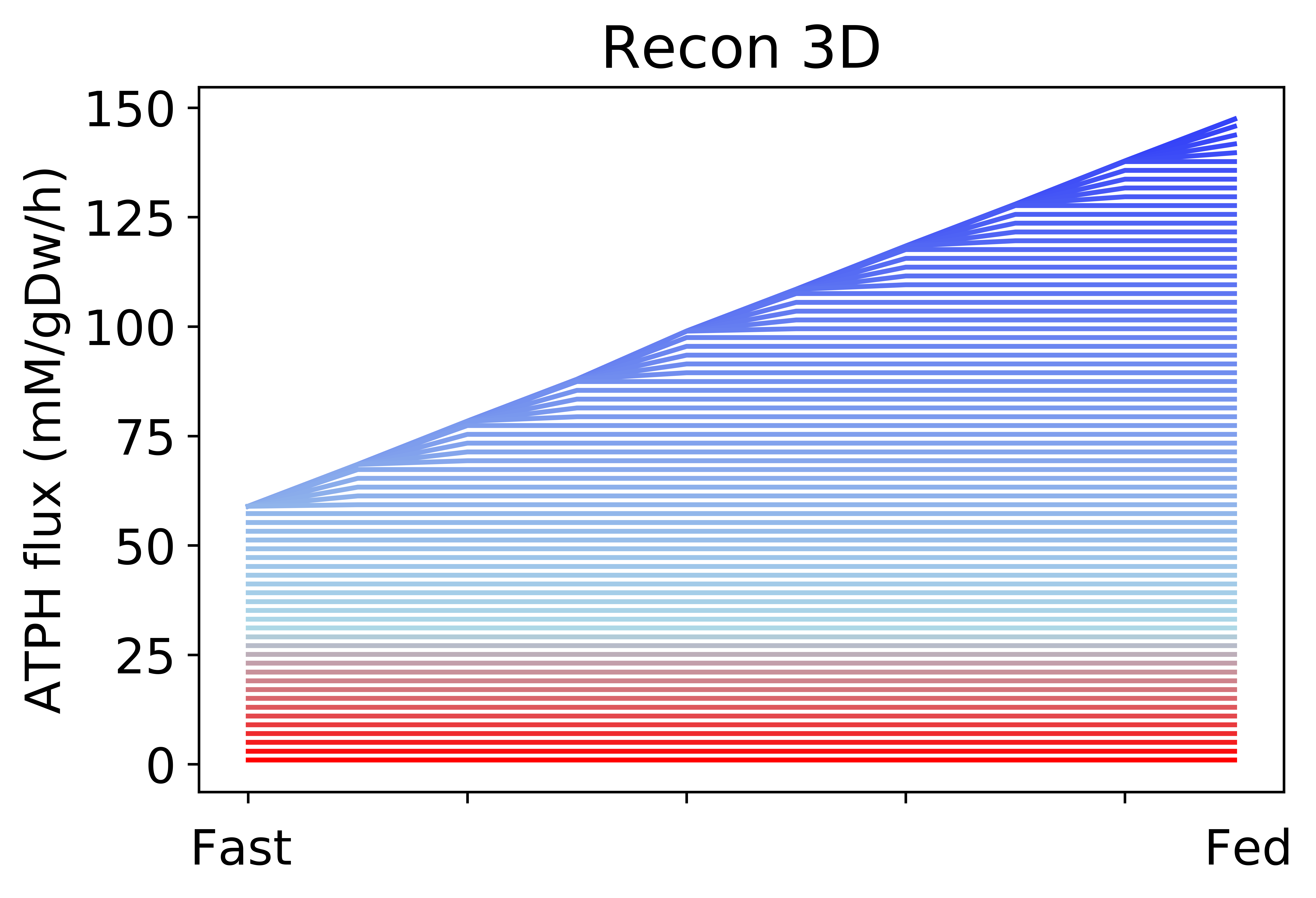

Supplement: Supplementary file 1 [file metabolites-11-00695-s001.zip › supplementary figures/figureS3B.png]

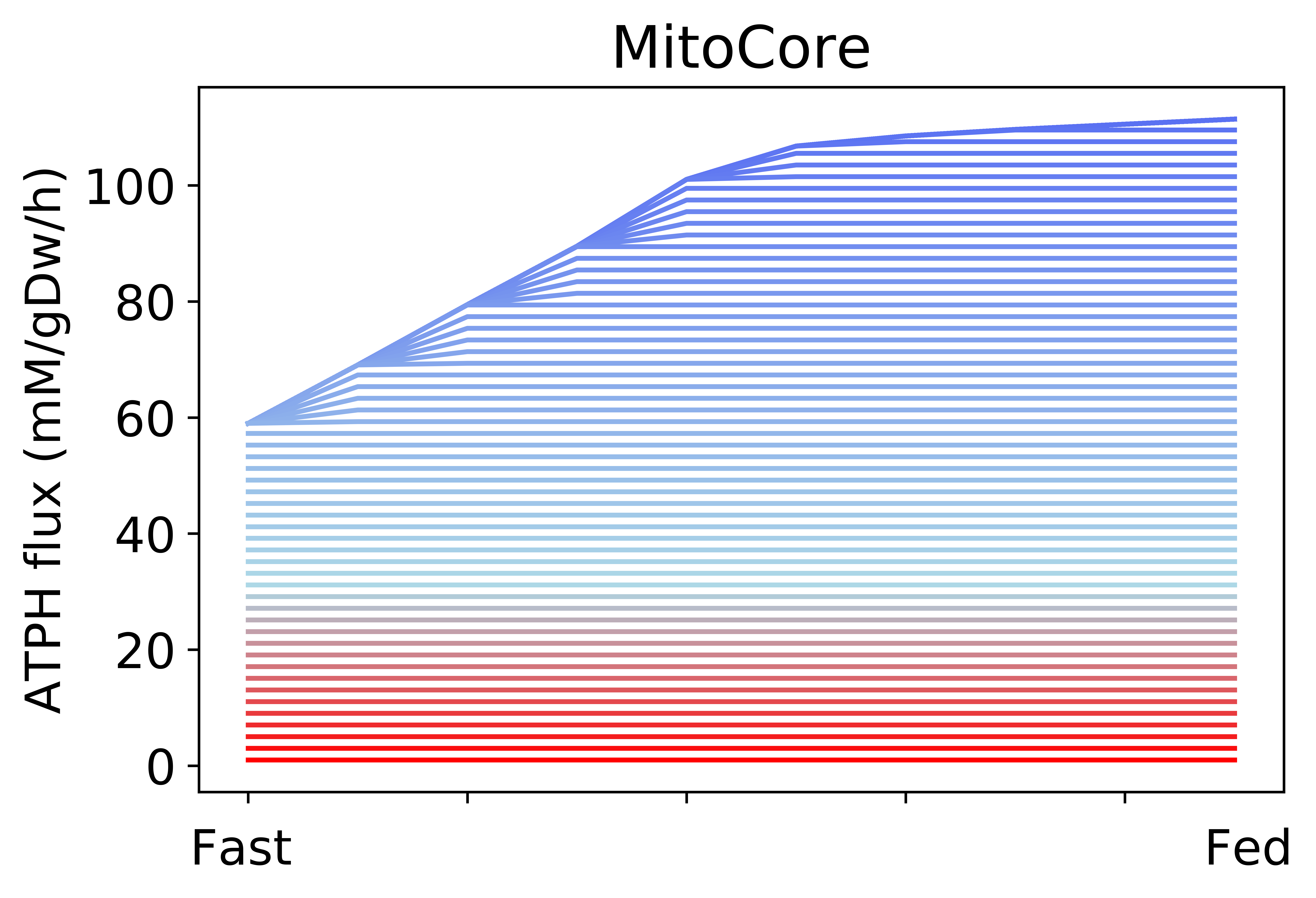

Supplement: Supplementary file 1 [file metabolites-11-00695-s001.zip › supplementary figures/figureS3C.png]

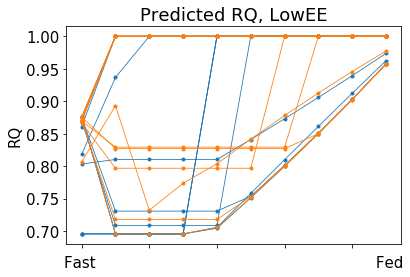

Supplement: Supplementary file 1 [file metabolites-11-00695-s001.zip › supplementary figures/figureS1.png]

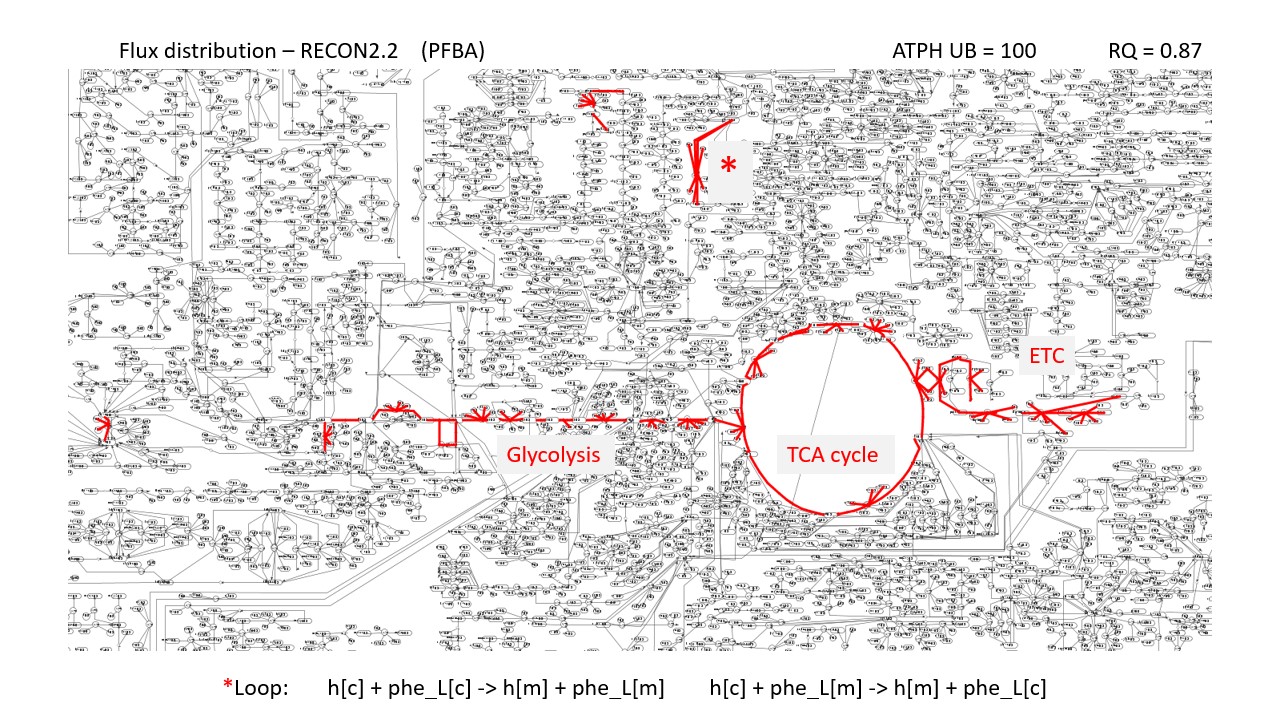

Supplement: Supplementary file 1 [file metabolites-11-00695-s001.zip › supplementary figures/figureS4A.jpg]

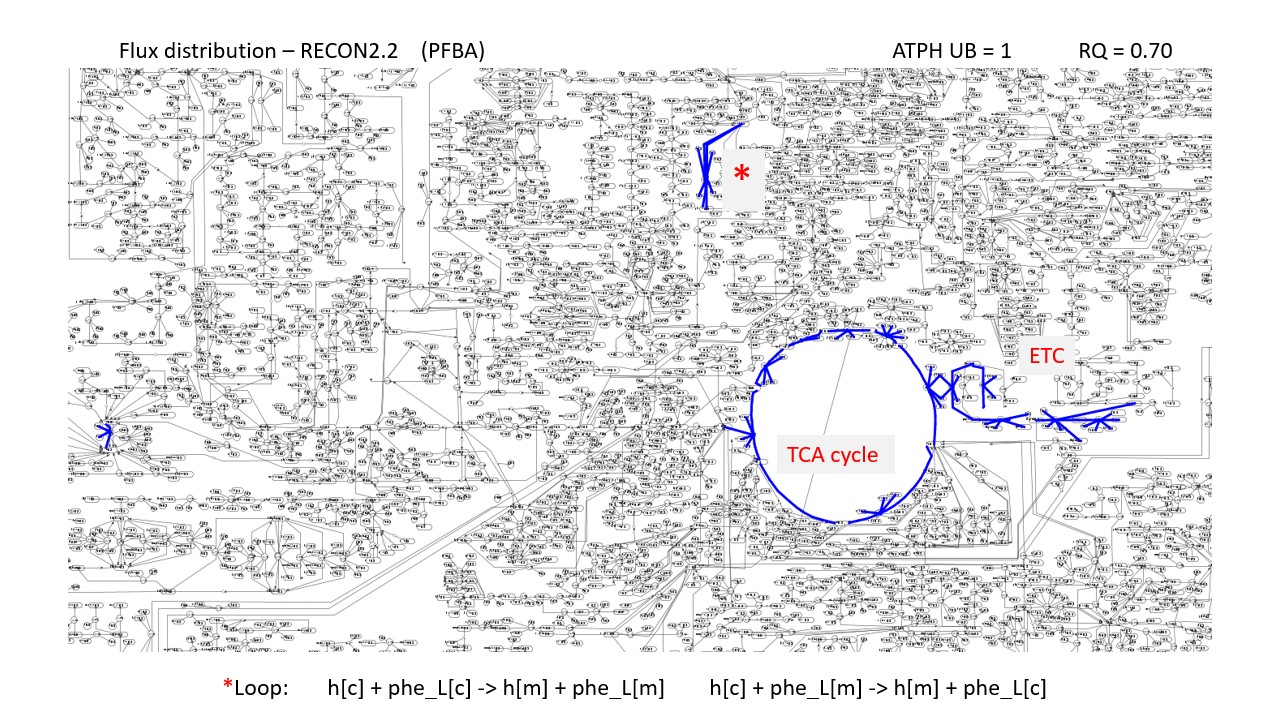

Supplement: Supplementary file 1 [file metabolites-11-00695-s001.zip › supplementary figures/figureS4B.jpg]

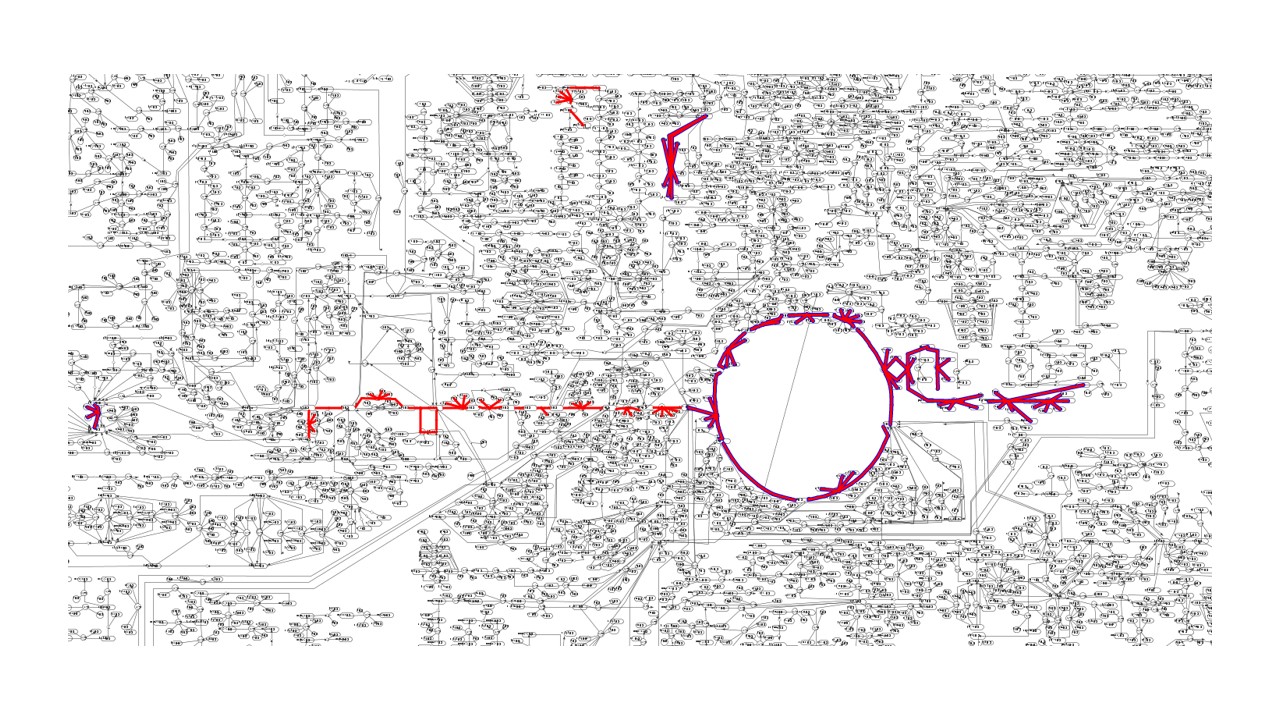

Supplement: Supplementary file 1 [file metabolites-11-00695-s001.zip › supplementary figures/figureS4C.jpg]
